# Transcriptomic analysis identifies candidate genes associated with striated muscle contraction during breast muscle development in meat-type ducks

**DOI:** 10.1016/j.psj.2026.107976

**Published:** 2026-10-17

**Authors:** Haoming Chang, Shuang Gu, Zhitong Li, Xin Wang, Shenhao Ma, Lianzhen Lu, Zihao Tang, Zhaoyu Geng, Sihua Jin

**Affiliations:** a College of Animal Science and Technology, Anhui Agricultural University, Hefei, 230036, China; b Anhui Province Key Laboratory of Local Livestock and Poultry, Genetic Resource Conservation and Germplasm Innovation, Hefei, 230036, China

**Keywords:** Embryonic myogenesis, Meat-type duck, Functional enrichment analysis, RNA-sequencing, Striated muscle contraction

## Abstract

The development of the breast muscle is critical for meat yield and quality in meat-type ducks. However, the transcriptional changes underlying breast muscle development in meat-type ducks remain poorly understood. In this study, we integrated phenotypic, histological, and myogenic regulatory factor analyses to characterize the developmental trajectory of the breast muscle, identifying embryonic day 19 (**E19**) as a pivotal transition point marked by peak muscle growth rate and maximal expression of *MYOD, MYOG*, and *MYF5*. Consequently, transcriptomic profiling of the pectoralis major muscle was conducted at three representative developmental stages, embryonic day 11 (**E11**), E19, and day of hatching (**D1**) using 15 cDNA libraries for RNA sequencing (**RNA-seq**). Pairwise comparisons identified 5,300, 5,167, and 8,393 differentially expressed genes (**DEGs**) in the E11 vs. E19, E19 vs. D1, and E11 vs. D1 comparisons, respectively. These DEGs were subsequently subjected to Gene Ontology (**GO**) and Kyoto Encyclopedia of Genes and Genomes (**KEGG**) enrichment analyses. Following the identification of common DEGs across the three developmental stages, protein-protein interaction (**PPI**) network analysis was integrated with gene set enrichment analysis (**GSEA**). This analysis revealed 24 common key genes involved in breast muscle development during meat-type duck growth, which converged our investigation specifically on the striated muscle contraction gene set. Furthermore, by integrating CytoHubba ranking and GSEA, we identified a core set of six candidate genes associated with striated muscle contraction during breast muscle development in ducks, namely *ACTN2, MYH11, MYL1, TNNC1, TNNI1*, and *TNNI2*. In conclusion, our findings delineate the dynamic transcriptomic landscape of duck breast muscle development and identify candidate genes associated with striated muscle contraction, which would be useful for future functional investigations of these genes and for developing molecular markers in ducks.

## Introduction

China is the world’s leading producer and consumer of meat-type ducks, generating over 3.5 billion ducks each year, which represents 75% of global meat-type duck production (Zhou et al., 2024). As a primary source of high-quality animal protein (Orounladji et al., 2022), skeletal muscle, particularly breast muscle, is one of the most economically significant components of poultry. Consequently, muscle mass accumulation is a pivotal trait that directly determines meat yield and quality (Chen et al., 2007). Crucially, in birds and mammals, the quantity of muscle fibers is predominantly established during the embryonic period and remains relatively constant after hatching or birth (Weng et al., 2025). Postnatal muscle growth mainly occurs through hypertrophy, which is facilitated by the fusion of satellite cells with existing muscle fibers (Kim et al., 2025). Therefore, characterizing skeletal muscle development during embryogenesis is essential for understanding the molecular basis of meat production.

Embryonic myogenesis is a highly orchestrated biological process involving the fusion of myoblasts to form multinucleated muscle fibers (Yasuda et al., 2023). Initially, myogenic progenitor cells undergo proliferation and differentiation into myotubes, which subsequently mature into functional myofibers (Kohn et al., 2023). This involves the coordinated regulation of several genes, transcription factors, and epigenetic mechanisms (Wang et al., 2024). The myogenic regulatory factor (**MRF**) family comprises core transcription factors that regulate the differentiation of myogenic precursor cells and is encoded by four genes: *MYOD, MYOG, MRF4*, and *MYF5* (Shen et al., 2024). *MYOD* and *MYF5* are responsible for the early directional differentiation and proliferation of myoblasts during embryonic development, whereas *MYOG* is essential for terminal differentiation and myotube formation (Olenic et al., 2025). Notably, functional muscle maturation extends beyond myofiber fusion, necessitating the coordinated assembly of the contractile apparatus. This transition is accompanied by coordinated changes in the expression of genes involved in striated muscle contraction and sarcomere organization, consistent with the maturation of nascent myotubes into functional muscle fibers (Luo et al., 2023).

In recent years, RNA sequencing (**RNA-seq**) has markedly advanced our understanding of the transcriptional profiles associated with skeletal muscle growth and maturation in livestock and poultry (Qin et al., 2024). Specifically, transcriptomic investigations have predominantly focused on performance-related traits, such as residual feed intake (Jin et al., 2025), meat quality parameters (San et al., 2021), and inter-breed growth discrepancies (Cao et al., 2023). Although these studies have provided valuable insights into metabolic regulation and extracellular matrix dynamics, the stage-specific transcriptional programs associated with functional assembly of the striated muscle contractile apparatus during late embryogenesis remain incompletely characterized.

In the present study, we aimed to identify the regulatory genes and signaling pathways associated with breast muscle development in meat-type ducks. We performed phenotypic, histological, myogenic-marker, and transcriptomic profiling of the pectoralis major muscle across key embryonic developmental stages and integrated differential expression analysis, functional enrichment, protein–protein interaction (**PPI**) network analysis, gene set enrichment analysis (**GSEA**) and hub-gene screening. Particular attention was given to the striated muscle contraction gene set to identify candidate genes associated with breast muscle development. This study provides a stage-resolved, phenotype-linked characterization of embryonic breast muscle development in meat-type ducks and identifies key candidate genes for future functional investigation.

## Materials and methods

### Ethics statement

All animal experiments were carried out in accordance with the Guidelines for the Care and Use of Experimental Animals established by the Ministry of Agriculture of China (Beijing, China). The whole protocols were approved by the Institutional Animal Care and Use Committee of Anhui Agricultural University (permit number: SYXK 2016-007), and all efforts were made to minimize experimental animal suffering.

### Experimental animals and phenotypic determination

All meat-type ducks utilized in this research were obtained from a random mating population supplied by Huangshan Qiangying Duck Breeding Co., Ltd. (Huangshan, China). The meat-type ducks were developed from genetically unrelated sources and had undergone selection as a closed breeding population for 12 generations. Fertilized half-sibling eggs of the meat-type ducks were incubated under standardized conditions (37.8°C, 60% relative humidity, with continuous ventilation). Embryos were collected on embryonic days 11 (**E11**), E15, E19, E23, and E27, and on the day of hatching (**D1**). The breast muscles from both sides of the embryos were separated at each time point. The whole embryo body and breast muscle were weighed to accurately calculate the breast muscle weight ratio. The left and right pectoralis major muscles were sampled for RNA extraction and myofiber morphology analysis, respectively. *MYOD, MYOG*, and *MYF5* expression at different developmental stages was also determined. Primer sequences are provided in Supplementary Table S1.

Fifteen male embryos were selected via molecular sex identification (five biological replicates per stage at E11, E19, and D1). The sex-linked *CHD1* gene was amplified for molecular sex identification (Van Der Hofstadt et al., 2025). The forward and reverse primers for this gene were 5′-TGCAGAAGCAATATTACAAGT-3′ and 5′-AATTCATTATCATCTGGTGG-3′, respectively (Purwaningrum et al., 2019). The PCR products were separated using agarose gel electrophoresis. Male embryos displayed a single band at approximately 500 bp, whereas females exhibited two bands at approximately 350 and 500 bp (Supplementary Fig. S1). Only samples from genetically confirmed male embryos were used for downstream RNA-seq and gene expression analyses.

### Muscle fiber morphological characteristics

Breast muscle samples were fixed in 4% paraformaldehyde for 48 h, dehydrated using graded ethanol solutions, and embedded in paraffin. The wax blocks were sectioned transversely to a thickness of 10 µm using a microtome. The sections were deparaffinized and stained with haematoxylin and eosin. The stained sections were scanned using a high-resolution digital slide scanner (Pannoramic MID; 3DHISTECH, Budapest, Hungary) to generate whole-slide images. Five random fields containing muscle fibers from each whole-slide image were selected and imported into ImageJ software (v1.53) to measure the myofiber diameter and density of muscle fibers.

### RNA library preparation and sequencing

Total RNA was extracted from each pectoralis major muscle using TRIzol reagent (Invitrogen Life Technologies, Thermo Fisher Scientific, Carlsbad, CA, USA), according to the manufacturer’s instructions. RNA integrity was assessed through agarose gel electrophoresis, and purity was determined using a NanoDrop 2000 spectrophotometer (Thermo Fisher Scientific, Waltham, MA, USA).

We used mRNA capture beads to extract mRNA from total RNA. After purification, the mRNA underwent fragmentation at an elevated temperature. This fragmented mRNA served as a template in a reverse transcription enzyme mixture for the synthesis of first-strand cDNA. During the synthesis of the second strand of cDNA, end repair and A-tailing steps were carried out. Subsequently, adapters were ligated, and purification to isolate target fragments was conducted using Hieff NGS DNA selection beads. The library was amplified using PCR and sequenced on an Illumina HiSeq 4000 platform at Gene Denovo Biotechnology Co. Ltd. (Guangzhou, China).

### Data quality control and differential gene expression analysis

The evaluation of raw data quality was conducted with FastQC (v0.11.9; http://www.bioinformatics.babraham.ac.uk/projects/fastqc/). Using Cutadapt software (v4.1), adapter sequences and low-quality reads were eliminated. The clean reads were aligned to the duck reference genome [NCBI_GCF_015476345.1] using TopHat2 (v2.0.3.12) and mapped using Bowtie2 (v2.5.1). Read alignments and genomic positioning were inferred based on splice-junction mapping. Aligned reads from every sample were assembled using StringTie (v1.3.1) with a reference-guided approach. Gene expression levels were measured as transcripts per kilobase of exon model per million mapped reads, calculated using RSEM (v1.2.28) to normalize for both gene length and sequencing depth.

Differential gene expression between developmental stages was evaluated using the edgeR package (v3.24) in R. Genes were considered differentially expressed with a threshold of |log_2_FC| ≥ 1 and false discovery rate < 0.05. Positive and negative log_2_FC values indicated upregulation and downregulation, respectively.

### Functional enrichment analysis

Gene Ontology **(GO**) and Kyoto Encyclopedia of Genes and Genomes (**KEGG**) pathway analyses were performed using the OmicSmart Cloud Platform (http://www.omicsmart.com/). Enrichment analyses were conducted to screen for biological processes, molecular functions, cellular components, and related signaling pathways that were significantly enriched in the DEGs. Significance was set at FDR ≤ 0.05.

### Gene set enrichment analysis

Gene set enrichment analysis (**GSEA**) was performed to identify biological processes and pathways. For each pairwise comparison among E11, E19 and D1, all expressed genes were ranked according to their differential expression levels. Predefined GO terms and KEGG pathways were used as reference gene sets. The normalized enrichment score (**NES**) was used to assess enrichment strength, and its direction was interpreted according to the ranking order of each comparison. Gene sets with |NES| > 1 and nominal *P* < 0.05 were considered significantly enriched.

### Key gene network analysis

Focusing on the overlapping DEGs identified across the three pairwise comparisons, we constructed a PPI network using the STRING database (http://string-db.org) to explore their potential functional connectivity. For species supported by the database, the DEG datasets were uploaded and mapped to their corresponding protein entries. The resulting PPI networks were imported into Cytoscape (v3.8.2) for visualization and topological analysis. Hub gene prioritization was performed using CytoHubba within Cytoscape. The top 10 genes were independently ranked using six centrality measures: Maximal Clique Centrality (**MCC**), Maximum Neighborhood Component (**MNC**), Degree, Edge Percolated Component (**EPC**), Bottleneck, and Closeness. Genes ranked in the top 10 by each of the six algorithms were defined as consensus network candidates. To further refine these candidates, they were intersected with the genes contributing to the enrichment of the muscle development-related gene sets identified by GSEA. Genes shared by both sets were designated as the final candidate hub genes.

### Quantitative real-time PCR

Qualified RNA was used as a template for reverse transcription using the EasyScript One-Step gDNA Removal and cDNA Synthesis Super Mix kit (TransGen Biotech, Beijing, China), according to the manufacturer’s protocol. Nine DEGs were selected for quantitative real-time PCR (**qPCR**) validation. Gene-specific primers were designed using Primer Premier (v5.0) (Supplementary Table S1). qPCR was performed on an ABI 7500 Real-Time PCR System (Applied Biosystems Inc., Foster City, CA, USA) using NovoStart SYBR qPCR Super Mix Plus (Novoprotein Scientific, Shanghai, China). Duck *β*-actin was used as the internal reference gene, with relative gene expression calculated using the 2^−ΔΔCT^ method (Livak and Schmittgen, 2001; Wu et al., 2022). Each reaction was performed in triplicate.

### Statistical analysis

A one-way analysis of variance, accompanied by Duncan’s post hoc test using SPSS (v26.0, IBM Corp., Armonk, NY, USA), was employed to assess the phenotypic data and gene expression levels across developmental stages. Results are shown as the mean ± standard deviation. Statistically, *P* < 0.01 is defined as a highly significant difference, whereas *P* < 0.05 represents a significant difference.

## Results

### Characterization of breast muscle and gene expression changes during different embryonic development stages

A graphical depiction of the experimental design and workflow is provided in Fig. 1a. We measured body weight, breast muscle weight, and breast muscle weight ratio and examined the muscle fiber morphology of meat-type ducks at various embryonic developmental stages (E11, E15, E19, E23, E27, and D1). The results revealed that the body weight of the embryos increased steadily during the developmental period (Fig. 1b). The weight of the breast muscle increased continuously from E11 to E23 but remained relatively stable from E23 to hatching (Fig. 1c). The breast muscle weight first increased and then decreased over time, peaking at E19 (Fig. 1d). Histological characteristics (Fig. 1e) showed that the diameter of the muscle fibers progressively increased with development (Fig. 1f), whereas fiber density showed a declining trend over time (Fig. 1g).

**Fig. 1:**
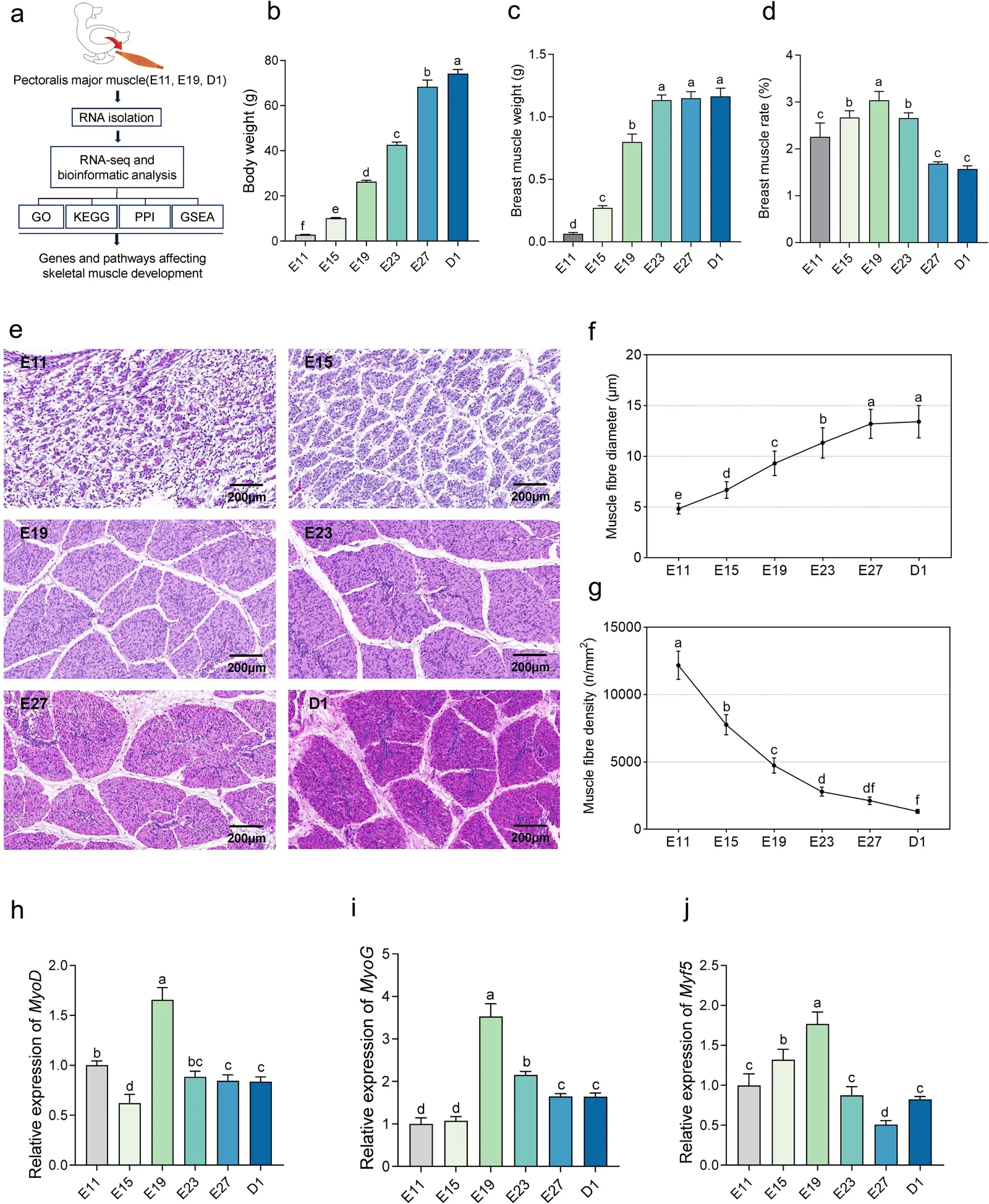
Characterization of the breast muscle and gene expression changes during different developmental stages. (a) Experimental paradigm for transcriptome profiling of the breast muscle at three embryonic developmental stages in meat-type ducks. (b) Changes in body weight on embryonic day 11 (E11), E15, E19, E23, E27, and day of hatching (D1). (c) Changes in breast major muscle weight at E11, E15, E19, E23, E27, and D1. (d) Changes in breast major muscle ratio at E11, E15, E19, E23, E27, and D1. (e) Haematoxylin and eosin staining (200 ×). (f) Muscle fiber diameter. (g) Muscle fiber density. (h) Relative mRNA expression patterns of *MYOD* at different time points. (i) Relative mRNA expression patterns of *MYOG* at different time points. (j) Relative mRNA expression patterns of *MYF5* at different time points. Different superscripts on the bars indicate significant differences (*P* < 0.05).

Relative gene expression analysis showed that *MYOD* expression initially decreased before increasing and then finally declined to a stable level, with the highest relative expression observed at E19 (Fig. 1h). *MYOG* expression gradually increased, peaked at E19, and subsequently decreased before reaching a plateau (Fig. 1i). *MYF5* expression exhibited a biphasic increase, with an initial increase followed by a transient decline and a second increase, reaching a peak at E19 (Fig. 1j). Based on these results, E11, E19, and D1 were selected for RNA-seq to represent the early developmental stage, the transitional stage associated with peak breast muscle rate and maximal myogenic marker expression, and the hatching stage, respectively.

### Descriptive statistics of RNA-seq data

All samples in this study generated high-quality clean data, with clean read percentages exceeding 97%. The Q20 and Q30 quality scores were greater than 96.31% and 89.96%, respectively, indicating a high base-calling accuracy. The GC content was consistently stable across the samples, ranging from 48.02% to 52.91%. The proportion of multi-mapped reads varied between 6.96% and 11.70%, whereas the total number of mapped reads exceeded 82.30% for all samples. Further annotation analysis showed that 82.77–86.36% of the mapped reads were aligned to exon regions, demonstrating high specificity of transcriptome sequencing (Supplementary Table S2).

### Analysis of differentially expressed genes

Principal component analysis (**PCA**) revealed high biological reproducibility within the sample groups and clear separation between the groups (Fig. 2a). We examined the overlap of DEGs across pairwise comparisons. A total of 1,549 genes were differentially expressed in all three comparisons (Fig. 2b). A total of 5,300 DEGs were identified between E11 and E19. Among them, 1,189 genes exhibited upregulation and 4,111 showed downregulation at E19 relative to those at E11 (Fig. 2c). The comparison between E19 and D1 identified 5,167 DEGs, including 1,520 upregulated and 3,647 downregulated genes at D1 compared to those at E19 (Fig. 2d). Similarly, the E11 vs. D1 comparison identified 8,393 DEGs, comprising 6,854 upregulated and 1,539 downregulated genes at D1 relative to E11 (Fig. 2e).

**Fig. 2:**
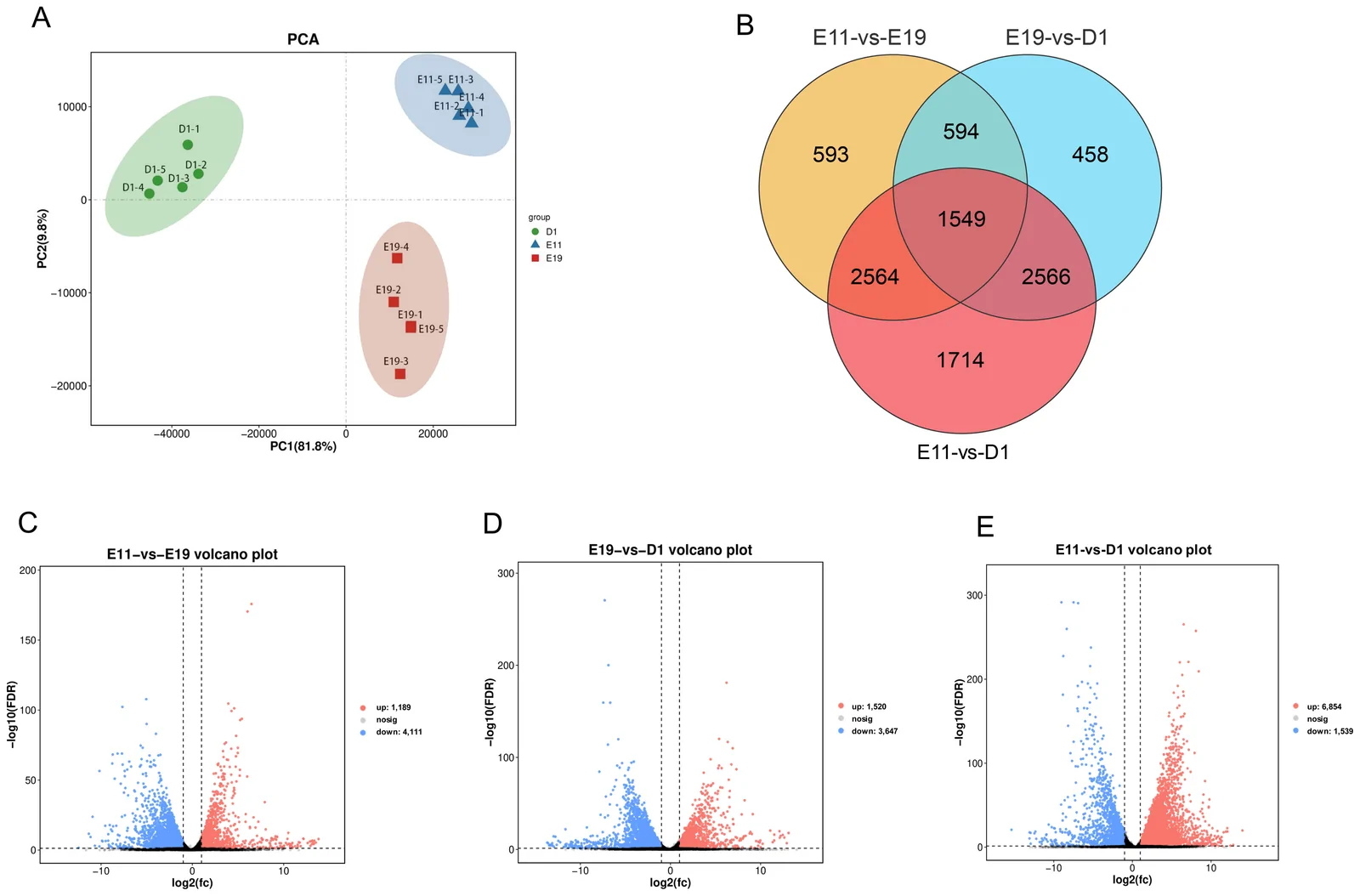
Analysis of differentially expressed genes. (a) Principal component analysis of RNA-seq data from duck embryo pectoralis major muscle tissue. (b) Venn diagram of differentially expressed genes (DEGs). (c) Volcano plot of DEGs between E11 and E19 (E11 vs. E19) in pectoralis major muscle tissue. Red dots denote markedly upregulated genes, blue dots denote markedly downregulated genes (|log_2_FC| ≥ 1 and false discovery rate < 0.05), and black dots indicate non-significant DEGs. (d) Volcano plot of DEGs between E19 and D1 (E19 vs. D1) in pectoralis major muscle tissue. (e) Volcano plot of DEGs between E11 and D1 (E11 vs. D1) in pectoralis major muscle tissue.

### Enrichment analysis of all differentially expressed genes

Hierarchical clustering of DEGs across different stages (E11, E19, and D1) revealed distinct gene expression patterns, clearly separating the samples into their respective groups based on developmental time points (Supplementary Fig. S2). In the E11 vs. E19 comparison, GO enrichment results showed that DEGs were mainly associated with system development, developmental process, single-organism developmental process, anatomical structure development, multicellular organismal process, and multicellular organism development (Fig. 3a and b). For the E19 vs. D1 comparison, the GO cellular component terms primarily included the extracellular region, extracellular matrix, supramolecular fiber, synaptic membrane, postsynaptic membrane, and cytoskeleton (Fig. 3c and d). In the E11 vs. D1 group, GO molecular function analysis revealed significant enrichment in binding, cytoskeletal protein binding, and protein binding (Fig. 3e and f).

**Fig. 3:**
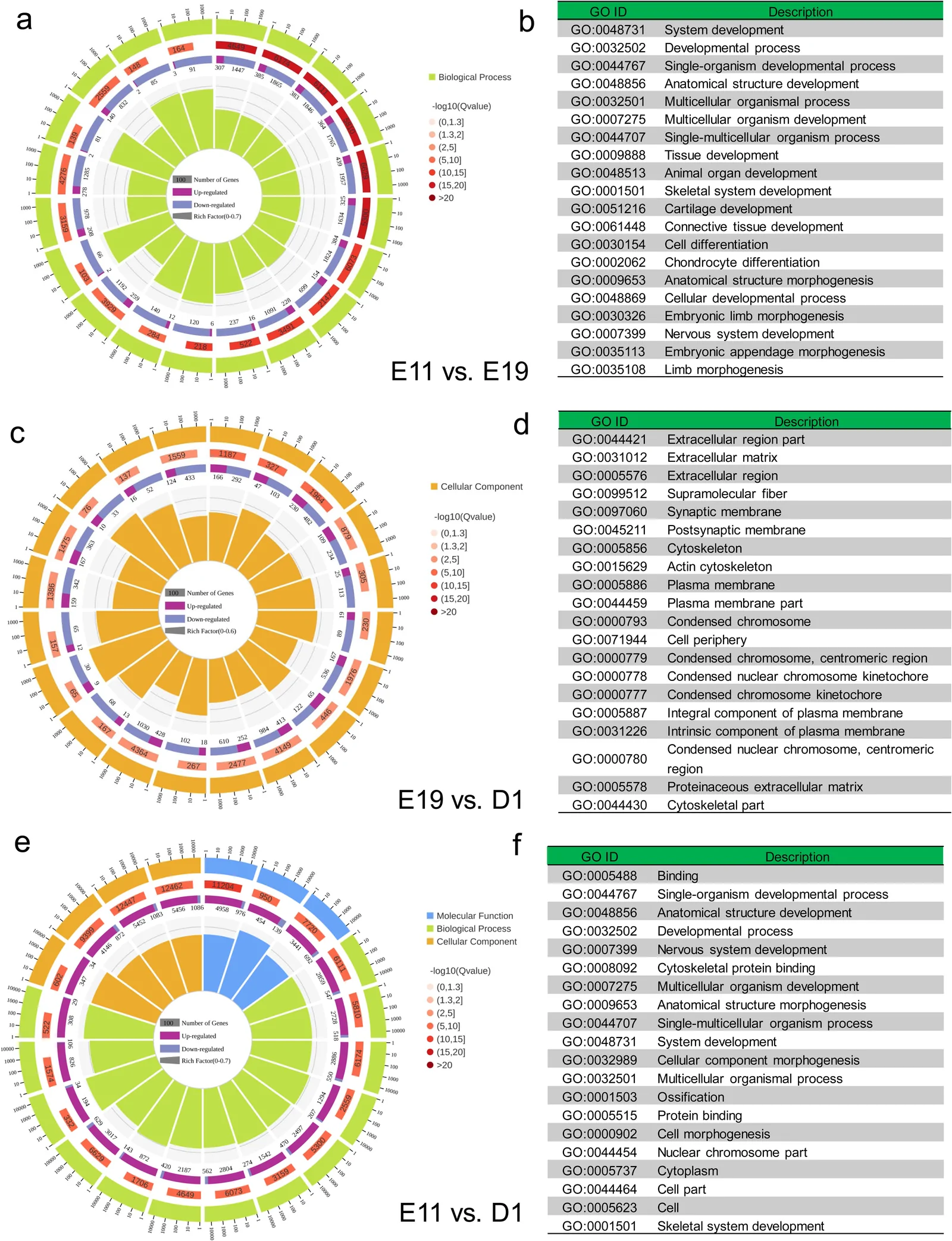
Gene ontology (GO) enrichment analysis of differentially expressed genes. The top 20 significantly enriched GO terms are shown in circular diagrams for E11 vs. E19 (a), E19 vs. D1 (c), and E11 vs. D1 (e); and the top 20 biological process terms are shown for E11 vs. E19 (b), E19 vs. D1 (d), and E11 vs. D1 (f).

KEGG pathway analysis across E11, E19, and D1 revealed that the Wnt, PI3K-Akt, and Hippo signaling pathways were significantly different during the early and mid-stages of pectoralis major muscle development (Fig. 4a). The ECM–receptor interaction and cell cycle pathways were significantly enriched across all three stages, whereas the adherens junction pathway showed significant enrichment, specifically in the early stages of pectoralis major muscle development (Fig. 4b and c; Supplementary Tables S3–5).

**Fig. 4:**
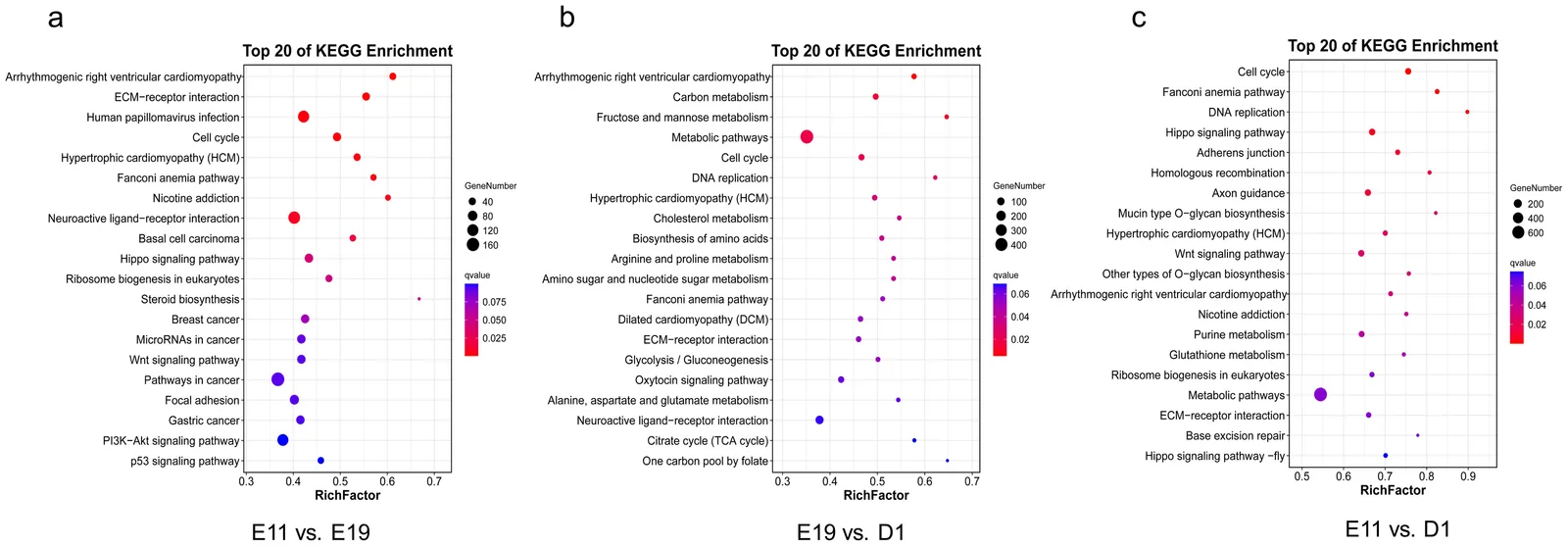
Significantly enriched Kyoto Encyclopedia of Genes and Genomes (KEGG) pathways of differentially expressed genes. The top 20 significantly enriched KEGG pathways are shown in bubble charts-for E11 vs. E19 (a), E19 vs. D1 (b), and E11 vs. D1 (c).

### Protein–protein interaction network analysis

Protein–protein interaction (**PPI**) networks were constructed for the DEGs identified in the three developmental comparisons. The E11 vs. E19 PPI network contained 78 nodes and 748 edges, with key genes including *CAV3, TNNC2,* and *TNNI2* (Fig. 5a). The E19 vs. D1 PPI network comprised 74 nodes and 406 edges, with *NCK2, RAC3,* and *MAPK11* identified as key genes (Fig. 5b). The PPI network of the E11 vs. D1 comparison contained 88 nodes and 674 edges, with *CTNNB1, TPM2, EGF, TRRAP,* and *TGFB3* identified as key genes (Fig. 5c). Overlap analysis of the nodes from the three PPI networks identified 24 common genes, including *ACTN2, MYH11, MYL1, TNNC1, TNNI1, TNNI2, MYOZ1, NEB*, and *TNNT2*, among others. Functional enrichment analysis of these common genes using the STRING database revealed significant associations with striated muscle contraction, TGF-beta signaling, and fibroblast growth factor receptor signaling. Among these enriched terms, a total of 11 genes were associated with the striated muscle contraction gene set, indicating that this biological process may play an important role in breast muscle development (Fig. 5d).

**Fig. 5:**
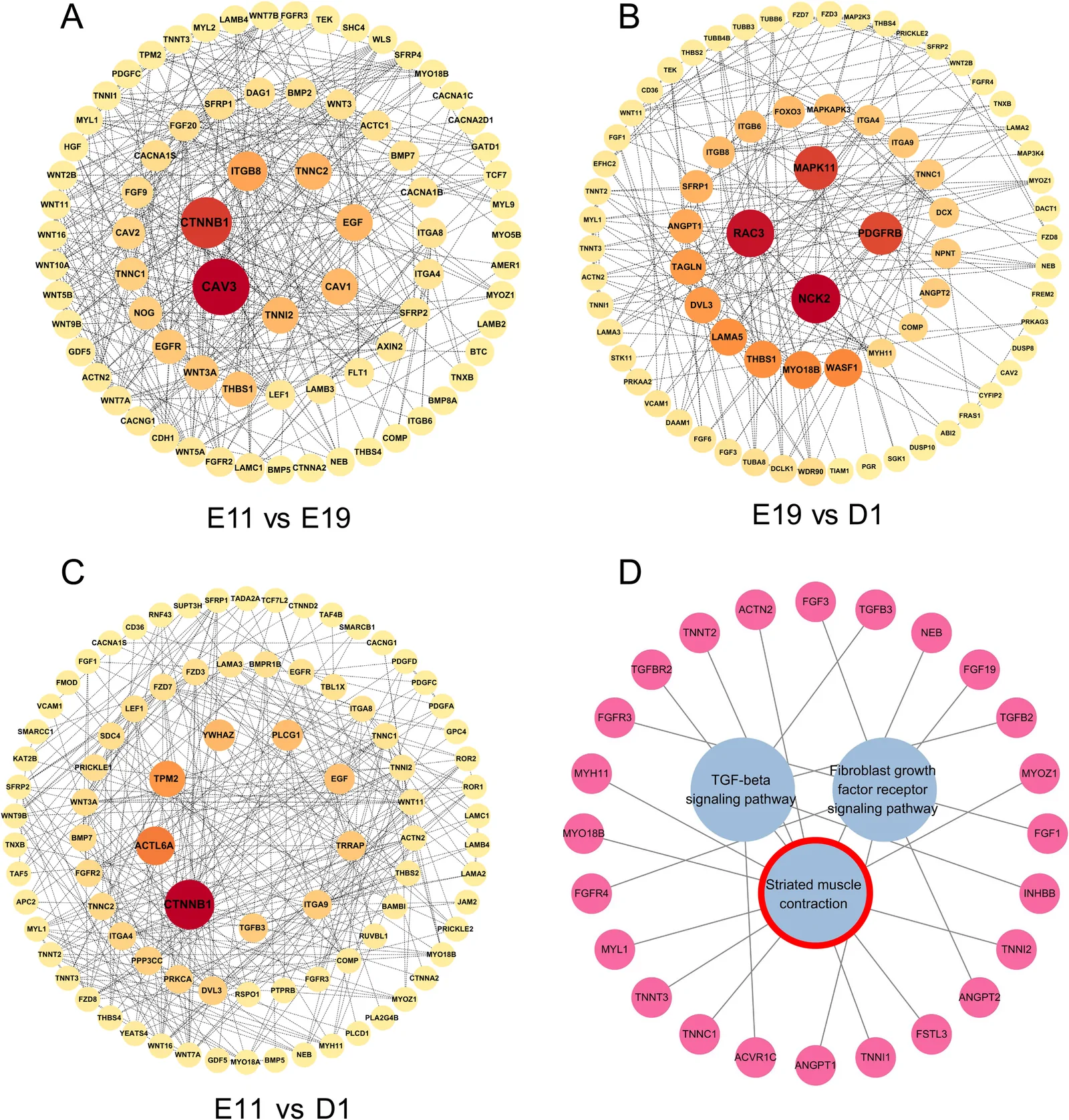
Protein–protein interaction (PPI) network analysis of differentially expressed genes at (a) E11 vs. E19, (b) E19 vs. D1, and (c) E11 vs. D1. Node size and color indicate node degree; larger and darker-red nodes represent genes with more interaction partners. (d) Screening of common differentially expressed genes at E11, E19, and D1. Pink nodes represent genes, blue nodes represent enriched gene sets.

### Gene set enrichment analysis

To further support the functional relevance of these common genes, gene set enrichment analysis (**GSEA**) was performed to evaluate muscle-related biological processes across the developmental stages. Three significantly enriched gene sets were identified, including muscle cell development, structural constituents of muscle, and skeletal muscle contraction (*P* < 0.05). The key genes contributing to muscle cell development were *ACTN2* and *MYH11* (Fig. 6a). The structural constituents of muscle were primarily driven by *ACTN2, MYH11,* and *MYL1* (Fig. 6b). Furthermore, the skeletal muscle contraction was significantly enriched, featuring key contributing genes, including *TNNC1, TNNI1,* and *TNNI2* (Fig. 6c). These six genes were also mapped to the striated muscle contraction gene set, further supporting the importance of contractile apparatus-related processes during embryonic breast muscle development.

**Fig. 6:**
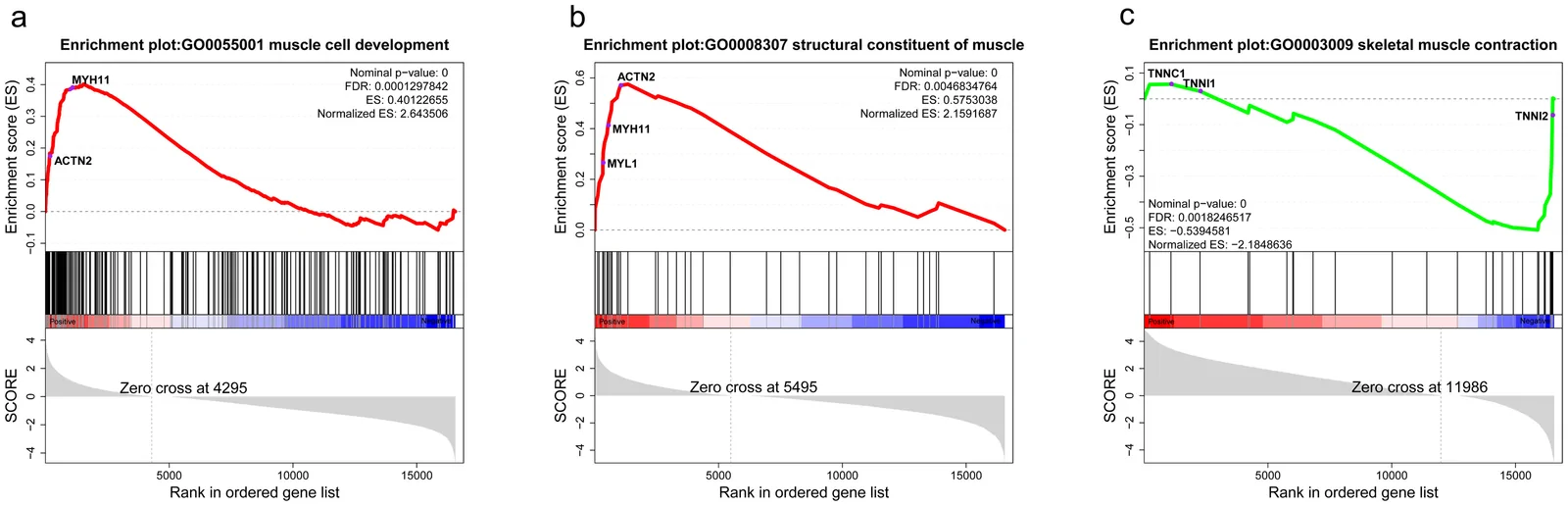
Gene set enrichment analysis plots of genes involved in various biological processes at different embryonic time points. Three significantly enriched gene sets were identified (*P* < 0.05). The analysis showed that DEGs across the E11, E19, and D1 groups were primarily related to muscle cell development (a), structural constituents of muscle (b), and skeletal muscle contraction (c).

### Identification of key candidate genes associated with the striated muscle contraction gene set

To further prioritize candidate hub genes from the 24 common genes among the three comparison groups, we constructed a PPI network (Fig. 7a). Based on PPI network analysis, 11 genes were associated with the striated muscle contraction gene set (Fig. 7b). CytoHubba analysis was then performed using six topological algorithms, including MCC, MNC, Degree, Bottleneck, Closeness, and EPC. The intersection of the top 10 genes across these algorithms yielded nine candidate hub genes (Table 1). To assess the concordance between the RNA-seq and qPCR results, the nine genes were selected for qPCR validation. The expression profiles across the three developmental stages were generally consistent with the RNA-seq data, supporting the reliability of the transcriptomic results (Fig. 7c). To further characterize these candidate genes, we examined their expression patterns across the three developmental stages. As shown in Fig. 7d, the expression levels of *ACTN2, MYH11, MYL1*, and *TNNI2* progressively increased and reached a peak at D1. Notably, *TNNC1* and *TNNI1* displayed distinct temporal patterns, with marked upregulation at E19 followed by a pronounced decline at D1. In comparison, *SHC4, TNNT2* and *TGFB2* showed progressive downregulation from E11 to D1, reaching their lowest expression levels at D1 (Fig. 7d). Ultimately, by integrating CytoHubba ranking and GSEA results, *ACTN2, MYH11, MYL1, TNNC1, TNNI1*, and *TNNI2* were prioritized as final key candidate genes associated with striated muscle contraction. Furthermore, PPI network analysis revealed that these six genes were highly interconnected, forming a densely interacting module (Fig. 7e).

**Fig. 7:**
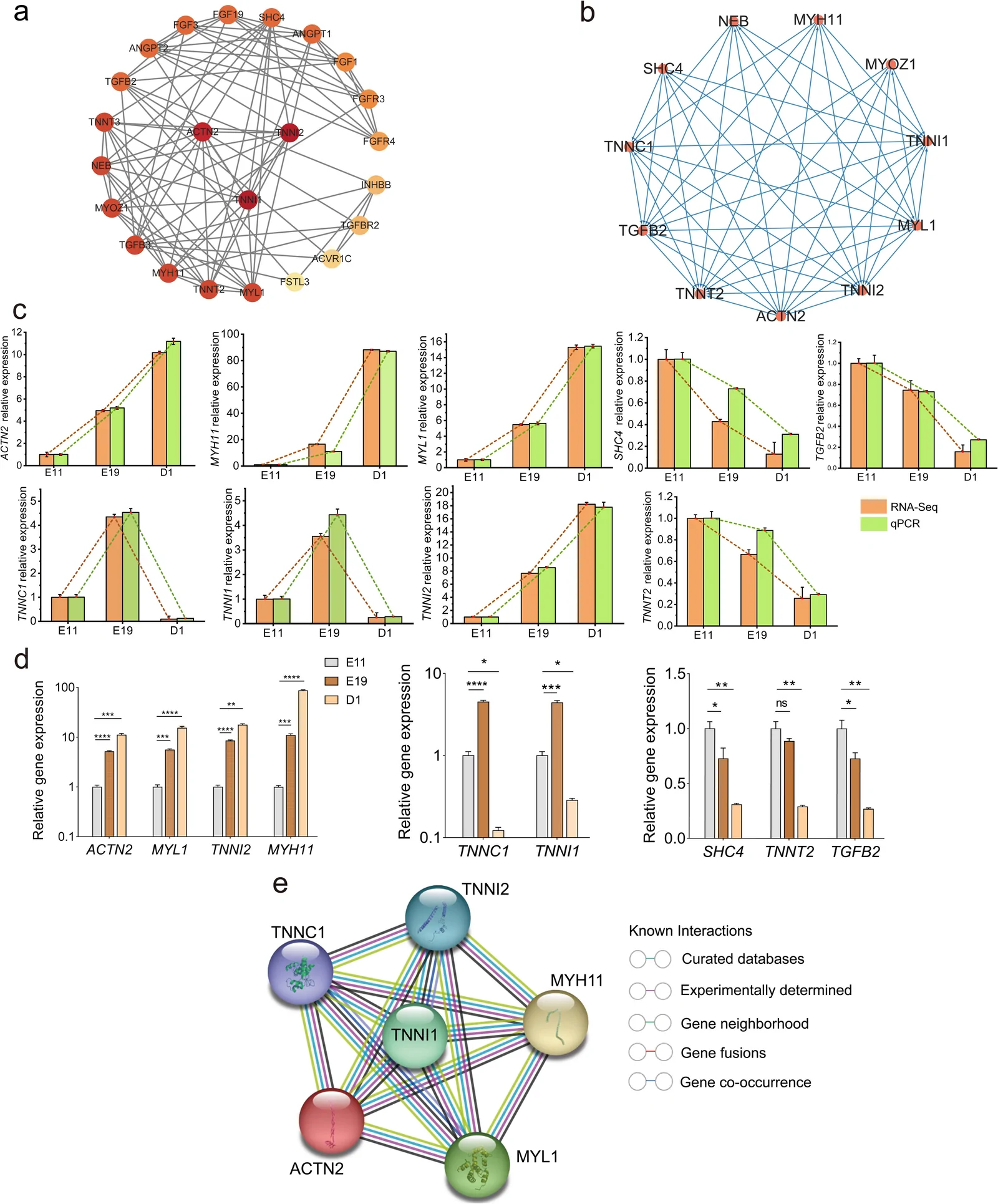
Identification of candidate genes enriched in the striated muscle contraction gene set. (a) PPI analysis of 24 key genes enriched in the common three comparisons. (b) Eleven genes were significantly associated with the striated muscle contraction gene set. (c) Validation of RNA-seq results by quantitative real-time PCR at E11, E19, and D1. (d) mRNA expression levels of hub genes at E11, E19, and D1. (e) PPI network of hub genes. All data are presented as mean ± SD. * indicates *P* < 0.05, ** indicates *P* < 0.01, *** indicates *P* < 0.001, and ns indicates *P* > 0.05.

**Table 1:** Top 10 genes ranked by six CytoHubba algorithms.

Rank	MCC	MNC	Degree	Bottleneck	Closeness	EPC
1	*TNNT2*	*TNNT2*	*TNNT2*	*ACTN2*	*TNNT2*	*MYL1*
2	*TNNI2*	*TNNI2*	*TNNI2*	*TNNT2*	*TNNI2*	*TGFB2*
3	*ACTN2*	*ACTN2*	*ACTN2*	*TNNI2*	*ACTN2*	*TNNI1*
4	*TNNI1*	*TNNI1*	*TNNI1*	*TNNC1*	*TNNI1*	*ACTN2*
5	*TGFB2*	*TGFB2*	*TGFB2*	*MYL1*	*TGFB2*	*TNNT2*
6	*MYL1*	*MYL1*	*MYL1*	*MYOZ1*	*MYL1*	*TNNC1*
7	*TNNC1*	*TNNC1*	*TNNC1*	*MYH11*	*TNNC1*	*TNNI2*
8	*NEB*	*NEB*	*NEB*	*SHC4*	*NEB*	*SHC4*
9	*MYH11*	*MYH11*	*MYH11*	*TNNI1*	*MYH11*	*MYH11*
10	*SHC4*	*SHC4*	*SHC4*	*TGFB2*	*SHC4*	*NEB*

## Discussion

The developmental trajectory of skeletal muscles during the late embryonic stage is highly dynamic and characterized by considerable physiological changes. A decline in the muscle fiber cross-sectional area and satellite cell mitotic activity was observed in the breast muscles of turkey during the late embryonic period (Moore et al., 2005). Similarly, breast muscle fiber atrophy occurs during late embryonic development in meat-type ducks (Chen et al., 2012). Our study revealed a critical transition in the development of breast muscle in ducks. The breast muscle weight of meat ducks increased until E23. The breast muscle ratio increased from E11 to E19 but showed a continuous downward trend from E19 to D1. Histological analysis further substantiated this timeline; myoblasts were predominantly single cells at E11, fused into multinucleated myotubes by E19, and matured into well-organized muscle fibers with an increased cross-sectional area by D1, consistent with previous morphological studies (Li et al., 2016). Collectively, these phenotypic and histological results suggest that E19 is a critical transition point, shifting from the phase of active myoblast proliferation to one dominated by myotube formation and maturation.

Members of the MRFs family act as master transcription factors in the early stages of myogenesis (Wang et al., 2022). MRFs influence this process by activating myogenic-specific gene programs while repressing non-myogenic lineages (Okamura et al., 2023; Wei et al., 2024). *MYF5* and *MYOD* are primarily responsible for committing progenitor cells to the myogenic lineage and can induce the trans-differentiation of other cell types, such as fibroblasts, into muscle cells (Ancel et al., 2024; Tan et al., 2023). Gene knockout experiments have shown that mice lacking both *MYOD* and *MYF5* are born completely devoid of skeletal muscle (Hu et al., 2023; Kim et al., 2023). *MYOG* functions in the later stages of myogenesis by promoting the fusion of myoblasts into myotubes and their subsequent maturation (Li et al., 2023). In the present study, the expression patterns of *MYOD, MYOG*, and *MYF5* provided molecular evidence supporting E19 as a critical developmental time. We observed that the mRNA levels of all three factors peaked concurrently at E19 before declining. Phenotypic and molecular data confirmed that E11, E19, and D1 represent key temporal landmarks in the development of the duck breast muscle, with myogenic differentiation peaking at E19.

Our functional enrichment analysis revealed that the DEGs were significantly enriched in several signaling pathways, including Wnt, PI3K-Akt, ECM–receptor interaction, and Hippo. These findings suggest that embryonic muscle development in ducks is regulated by a complex network rather than a single pathway. The Wnt and PI3K-Akt signaling pathways exert key regulatory functions during skeletal muscle development. For instance, the Wnt pathway facilitates myoblast fusion partly by regulating transcriptional activity and interacting with the Notch signaling pathway (Rudnicki and Williams, 2015). Similarly, the PI3K-Akt pathway is crucial for promoting myogenesis and cell survival because its activation is essential for processes such as muscle regeneration (Li et al., 2024). The ECM–receptor interaction and Hippo pathways regulate the structural and proliferative aspects of muscle formation. The ECM provides an essential scaffold for muscle development and influences muscle cell proliferation (Huang et al., 2023), differentiation, and function (Hu et al., 2022; Li et al., 2025). Enrichment of this pathway highlights the dynamic remodeling of the cellular microenvironment during myogenesis. The Hippo signaling pathway is a master regulator controlling tissue growth and organ size (Calses et al., 2019), and its activation and inactivation directly regulate cell proliferation. Therefore, its enrichment among the DEGs in this study likely reflects a tightly coordinated regulatory mechanism that first permits rapid myoblast expansion and then triggers cell cycle exit to enable terminal differentiation.

Among the common DEGs, six genes were identified as candidates associated with the striated muscle contraction gene set during breast muscle development. Among them, *ACTN2* encodes α-actinin-2, a cytoskeletal anchoring protein predominantly expressed in skeletal and cardiac muscles (Ranta-Aho et al., 2022). It plays a critical role in muscle regeneration by modulating focal adhesion signaling pathways, thereby regulating the activation and differentiation of muscle stem cells (Singer et al., 2021). Moreover, *MYH11* encodes myosin heavy chain 11, a key cytoskeletal component primarily expressed in smooth muscle cells (Takahashi et al., 2021). *MYH11* has been implicated in the regulation of muscle fiber composition (Yue et al., 2023), suggesting a broader influence on muscle development in livestock species. *MYL1* encodes myosin light chain 1, a critical regulatory subunit of the myosin complex. During skeletal muscle repair after injury, the upregulation of *MYL1* promotes satellite cell activation and muscle fiber remodeling (Ravenscroft et al., 2018).

The troponin complex, located on the thin filaments of striated muscles, serves as a calcium-sensitive molecular switch that regulates contraction (Sheng and Jin, 2016). In this study, we identified several key components of this complex, including *TNNC1, TNNI1*, and *TNNI2. TNNC1* encodes troponin C, a core regulatory protein for striated muscle contraction that binds calcium ions and promotes skeletal muscle myogenesis (Donkervoort et al., 2024; Li et al., 2022). Troponin I, represented by two isoforms identified here, *TNNI1* (slow-twitch) and *TNNI2* (fast-twitch), inhibits actin–myosin interactions in the absence of calcium (Huang et al., 2023; Mullen and Barton, 2000). During early myogenesis, *TNNI1* and *TNNI2* are co-expressed before their expression becomes restricted to slow and fast fibers, respectively (Xu et al., 2010).

Striated muscle contraction depends on the coordinated interaction between actin-containing thin filaments and myosin-containing thick filaments within sarcomeres (Brunello and Fusi, 2024), a process regulated by both thin-filament troponin–tropomyosin activity and thick-filament myosin availability (Lehman and Rynkiewicz, 2023). Importantly, emerging evidence supports an additional layer of thick-filament regulation. In resting muscle, a substantial fraction of myosin motors can be sequestered in inhibited conformations and must be recruited into an available pool during activation. This supports a dual-filament paradigm in which thin- and thick-filament activation are tightly coordinated through interfilament signaling pathways (Park-Holohan et al., 2021). At the tissue level, skeletal and cardiac muscle represent the two major striated muscle types and share a conserved contractile architecture, yet differ markedly in size and regenerative capacity (Shadrin et al., 2016).

Within this mechanistic framework, our transcriptomic analyses revealed significant enrichment of the striated muscle contraction gene set and consistently prioritized *ACTN2, MYH11, MYL1, TNNC1, TNNI1*, and *TNNI2* as central nodes. Together, these findings are consistent with coordinated maturation that integrates sarcomeric scaffolding (*ACTN2*), myosin-based force production (*MYH11, MYL1*), and troponin complex assembly (*TNNC1, TNNI1*, and *TNNI2*). Their stage-dependent expression patterns suggest that remodeling of the contractile machinery is closely associated with the transition from myoblast proliferation to muscle fiber maturation in meat-type ducks. Notably, although transcriptomic analyses have revealed six candidate genes associated with embryonic skeletal muscle development in meat-type ducks, their in vivo biological functions remain to be systematically validated. It has been emphasized that poultry muscle biology is at a critical juncture, transitioning from transcriptomic correlation to causal validation, with viral vectors and CRISPR/Cas9 genome editing tools providing powerful technological support for this shift (Gibril et al., 2025). Therefore, future studies are necessary to employ these functional genomics tools to systematically validate core candidate genes such as *ACTN2* and *MYL1* in vivo, thereby elucidating their precise roles in regulating breast muscle development and providing more reliable targets for molecular breeding in meat-type ducks.

## Conclusion

This study delineates the dynamic transcriptional landscape of breast muscle development in meat-type ducks and identifies E19 as a critical transition point associated with changes in breast muscle growth and myogenic marker expression. Integrated transcriptomic profiling, PPI network analysis, and GSEA highlighted the striated muscle contraction gene set as a molecular feature associated with breast muscle development. Notably, six hub genes, including *ACTN2, MYH11, MYL1, TNNC1, TNNI1*, and *TNNI2*, were prioritized as candidate genes associated with striated muscle contraction during breast muscle development. These findings provide new insights into the biological basis of embryonic breast muscle development in ducks. Further studies are required to investigate the functional roles of these genes in regulating breast muscle development of meat-type ducks.

## Availability of data and materials

The raw data were deposited in the Genome Sequence Archive (https://ngdc.cncb.ac.cn/gsa/) with the accession number CRA021331.

## Author contributions

Haoming Chang and Shuang Gu conceived and designed the study, performed sample collection and the transcriptome data analysis and interpretation, and drafted and revised the manuscript. Zhitong Li, Xin Wang, and Shenhao Ma designed and performed the experiments, collected samples at the different developmental stages, and critically revised the manuscript. Lianzhen Lu and Zihao Tang performed sample collection and project management, conducted the statistical analyses, and contributed to the writing and revision of the manuscript. Zhaoyu Geng conceptualized the project, contributed to the scientific evaluation and interpretation of the results, and critically revised the manuscript. Sihua Jin developed the overall study design, performed data collection, conducted the statistical analyses, critically reviewed and revised the manuscript, and provided funding support for data collection. All authors have read and approved the final version of the manuscript and agree to be accountable for all aspects of the work.

## Disclosures

The authors declare that they have no competing interests.

## Footnotes

Supplementary material associated with this article can be found, in the online version, at doi:10.1016/j.psj.2026.107976.
