# Supplementary material for: Transcriptomic analysis identifies candidate genes associated with striated muscle contraction during breast muscle development in meat-type ducks
Source: Poult Sci. 2026 Oct 17;105(12):107976. doi: 10.1016/j.psj.2026.107976 (PMC13738108; doi:10.1016/j.psj.2026.107976)
Supplement: Supplementary file 1 [file mmc2.pdf]

editage

Editing Certificate

Issued On May 04, 2026

This document certifies that the manuscript listed below has been edited to ensure language and grammar accuracy and is error free in these aspects. The edit was performed by professional editors at Editage, a brand of Cactus Technology Solutions Private Limited. The author's core research ideas were not altered in any way during the editing process. The quality of the edit has been guaranteed, with the assumption that our suggested changes have been accepted and the text has not been further altered without the knowledge of our editors.

QUALITY GUARANTEE • QUALITY GUARANTEE • QUALITY GUARANTEE •
THE editage PROMISE

Manuscript Title

Transcriptomic analysis identifies candidate genes associated with striated muscle contraction during breast muscle development in meat-type ducks

Authors

Haoming Chang, Shuang Gu, Zhitong Li, Xin Wang, Shenhao Ma, Lianzhen Lu, Zihao Tang, Zhaoyu Geng, Sihua Jin*

GAOJI_38_4

Job Code

Prabh Grewal, Senior Vice President, Editage

VERIFY AT
https://www.editage.cn/ecverify

VERIFICATION CODE
EC-261014-XFC24Q
