# Supplementary material for: Transcriptomic analysis identifies candidate genes associated with striated muscle contraction during breast muscle development in meat-type ducks
Source: Poult Sci. 2026 Oct 17;105(12):107976. doi: 10.1016/j.psj.2026.107976 (PMC13738108; doi:10.1016/j.psj.2026.107976)
Supplement: Supplementary file 2 [file mmc1.docx]

**Transcriptomic analysis identifies candidate genes associated with striated muscle contraction** **during breast muscle development in meat-type ducks**

Haoming Chang^a,b,1^, Shuang Gu^a,b,1^, Zhitong Li^a^, Xin Wang^a^, Shenhao Ma^a^, Lianzhen Lu^a,b^, Zihao Tang^a,b^, Zhaoyu Geng^a,b^, Sihua Jin^a,b,*^

^a^ *College of Animal Science and Technology, Anhui Agricultural University, Hefei 230036, China*

^b^*Anhui Province Key Laboratory of Local Livestock and Poultry, Genetic Resource Conservation and Germplasm Innovation, Hefei 230036, China*

^1^ These authors contributed equally to this work*.*

* Correspondence: Dr. and Associate Prof. Sihua Jin, College of Animal Science and Technology, Anhui Agricultural University, Hefei 230036, China.

Tel: +86-551-6578 6357

Fax: +86-551-6578 6328

E-mail: [jsh3235@126.com](mailto:jsh3235@126.com)

**Section: Genetics and Molecular Biology**

**Supplementary Table S1**

Primer sequences used for quantitative real-time PCR in meat-type ducks.

| Gene | Sequence (5’ to 3’) | Length (bp) | Annealing Temperature (℃) |
| --- | --- | --- | --- |
| *ACTN2* | F：CACAAGAGATGCCAAGGGTA | 86 | 57 |
|  | R：GTCCATTCTTCCTCCTGTCAA |  |  |
| *MYH11* | F：ACAGCTTCTACAGGCAAACC | 94 | 58 |
|  | R：RGCGGATGAATTTGCCGAATC |  |  |
| *MYL1* | F：GCACCAAAGAAGGACGTAAAG | 139 | 57 |
|  | R：CCTTGGAGAACTCGATCTTGA |  |  |
| *SHC4* | F：GGAGCGATAAAGAAGCGGAA  R：GGTTGGCAATAATCTGCTGTG | 154 | 58 |
| *TGFB2* | F：GCGCTACATTGACAGCAAAG | 112 | 58 |
|  | R：TCCAAGGTTCCTGTCTCTATGA |  |  |
| *TNNC1* | F：AGATCATGCTACAGGCAACC | 116 | 57 |
|  | R：GAACTCCAGGAACTCATCGT |  |  |
| *TNNI1* | F：CACAACACCAGGGAGATTAAGG | 165 | 58 |
|  | R：TTTCAGGTTGGCTCTGAGGT |  |  |
| *TNNI2* | F：CCGAGAAGCCCTTTTATACCT | 149 | 57 |
|  | R：GCCCTCCTCTTTTTCTCTTCA |  |  |
| *TNNT2* | F：TGGATTGAGGAAGAAGACGGT | 148 | 58 |
|  | R：GGTTTGGCATGAAGGGCTTG |  |  |
| *β-actin* | F：GCTATGTCGCCCTGGATTTC | 168 | 60 |
|  | R：CACAGGACTCCATACCCAAGAA |  |  |

Abbreviations: *ACTN2* = α-actinin-2; *MYH11* = myosin heavy chain 11; *MYL1* = myosin light chain 1; *SHC4* = Src homology 2 domain containing 4; *TGFB2* = transforming growth factor - beta 2; *TNNC1* = troponin C type 1; *TNNI1 =* troponin I1, slow skeletal muscle; *TNNI2,* troponin I2, fast skeletal muscle; *TNNT2* = troponin T2; *β-actin* = beta-actin.

F: forward primer; R: reverse primer.

**Supplementary Table S2**

Mapped statistical results of 15 libraries in meat-type ducks.

| Item | Raw reads (bp) | Clean reads (bp) | Q20 (%) | Q30 (%) | GC content (%) | Total mapped  reads (bp) | Unique mapped reads (bp) | Mapping rate (%) |
| --- | --- | --- | --- | --- | --- | --- | --- | --- |
| E11-1 | 6 256 698 600 | 6 161 262 507 | 97.49 | 93.00 | 48.37 | 41 413 680 | 34 120 618 | 89.40 |
| E11-2 | 5 673 610 200 | 5 555 848 332 | 97.31 | 92.55 | 48.02 | 37 554 522 | 31 036 176 | 89.88 |
| E11-3 | 7 889 569 500 | 7 731 484 479 | 96.48 | 90.40 | 48.38 | 52 111 988 | 42 330 267 | 88.19 |
| E11-4 | 6 642 551 100 | 6 551 445 669 | 97.45 | 92.89 | 48.28 | 43 991 712 | 36 211 222 | 89.43 |
| E11-5 | 5 726 853 300 | 5 613 532 524 | 97.30 | 92.50 | 48.20 | 37 927 154 | 31 150 737 | 89.55 |
| E19-1 | 6 956 553 300 | 6 841 754 796 | 97.33 | 92.56 | 50.27 | 45 925 268 | 36 514 767 | 87.70 |
| E19-2 | 5 616 406 200 | 5 505 395 105 | 97.32 | 92.57 | 49.01 | 37 181 646 | 29 916 422 | 89.02 |
| E19-3 | 6 454 326 900 | 6 319 540 099 | 96.69 | 91.23 | 52.91 | 42 468 864 | 31 451 915 | 82.30 |
| E19-4 | 6 717 670 500 | 6 586 707 359 | 96.94 | 91.74 | 48.68 | 44 378 928 | 35 674 838 | 88.74 |
| E19-5 | 6 970 608 300 | 6 809 483 626 | 97.24 | 92.45 | 49.37 | 46 119 456 | 37 488 484 | 88.92 |
| D1-1 | 5 904 180 300 | 5 812 402 445 | 97.31 | 92.52 | 50.00 | 39 004 236 | 29 209 970 | 86.59 |
| D1-2 | 7 997 200 800 | 7 829 473 717 | 96.54 | 90.65 | 50.29 | 52 701 848 | 38 312 354 | 83.98 |
| D1-3 | 8 001 493 800 | 7 820 293 628 | 96.32 | 90.14 | 50.50 | 52 724 396 | 38 342 299 | 84.22 |
| D1-4 | 8 046 711 600 | 7 854 672 337 | 96.31 | 89.96 | 51.12 | 53 077 540 | 38 181 815 | 83.38 |
| D1-5 | 7 305 662 700 | 7 146 584 660 | 97.20 | 92.34 | 50.59 | 48 282 174 | 35 699 757 | 85.35 |

Abbreviations: E11 = embryonic day 11; E19 = embryonic day 19; D1 = day of hatching.

Q20 represents the proportion of bases with a Phred quality score of > 20. Q30 represents the proportion of bases with a Phred quality score of > 30.

**Supplementary Table S3**

Top 20 pathways determined using the Kyoto encyclopedia of genes and genomes analysis in meat-type ducks (E11 vs. E19).

| Pathway ID | Pathways | Adjusted *P*-value | DEGs |
| --- | --- | --- | --- |
| ko05412 | Arrhythmogenic right ventricular cardiomyopathy (ARVC) | 1.48E-06 | 36 |
| ko04512 | ECM-receptor interaction | 2.15E-06 | 47 |
| ko05165 | Human papillomavirus infection | 2.76E-05 | 122 |
| ko04110 | Cell cycle | 2.91E-05 | 56 |
| ko05410 | Hypertrophic cardiomyopathy (HCM) | 4.52E-05 | 39 |
| ko03460 | Fanconi anemia pathway | 9.79E-05 | 29 |
| ko05033 | Nicotine addiction | 0.000124 | 24 |
| ko04080 | Neuroactive ligand-receptor interaction | 0.000162 | 135 |
| ko05217 | Basal cell carcinoma | 0.000397 | 31 |
| ko04390 | Hippo signalling pathway | 0.001167 | 62 |
| ko03008 | Ribosome biogenesis in eukaryotes | 0.001458 | 37 |
| ko00100 | Steroid biosynthesis | 0.001842 | 12 |
| ko05224 | Breast cancer | 0.002653 | 58 |
| ko05206 | MicroRNAs in cancer | 0.003646 | 61 |
| ko04310 | Wnt signalling pathway | 0.004067 | 59 |
| ko05200 | Pathways in cancer | 0.004333 | 170 |
| ko04510 | Focal adhesion | 0.004537 | 74 |
| ko05226 | Gastric cancer | 0.004885 | 59 |
| ko04151 | PI3K-Akt signalling pathway | 0.005924 | 117 |
| ko04115 | p53 signalling pathway | 0.00602 | 32 |

Abbreviation: DEGs = differentially expressed genes.

**Supplementary Table S4**

Top 20 pathways determined using the Kyoto encyclopedia of genes and genomes analysis in meat-type ducks (E19 vs. D1).

| Pathway ID | Pathways | Adjusted *P*-value | DEGs |
| --- | --- | --- | --- |
| ko05412 | Arrhythmogenic right ventricular cardiomyopathy | 1.57E-05 | 34 |
| ko01200 | Carbon metabolism | 7.74E-05 | 48 |
| ko00051 | Fructose and mannose metabolism | 0.000105 | 20 |
| ko01100 | Metabolic pathways | 0.00022 | 456 |
| ko04110 | Cell cycle | 0.000264 | 53 |
| ko03030 | DNA replication | 0.000471 | 18 |
| ko05410 | Hypertrophic cardiomyopathy | 0.000644 | 36 |
| ko04979 | Cholesterol metabolism | 0.000839 | 24 |
| ko01230 | Biosynthesis of amino acids | 0.000945 | 30 |
| ko00330 | Arginine and proline metabolism | 0.00128 | 24 |
| ko00520 | Amino sugar and nucleotide sugar metabolism | 0.00128 | 24 |
| ko03460 | Fanconi anemia pathway | 0.001935 | 26 |
| ko05414 | Dilated cardiomyopathy | 0.002022 | 38 |
| ko04512 | ECM-receptor interaction | 0.002226 | 39 |
| ko00010 | Glycolysis / Gluconeogenesis | 0.002299 | 27 |
| ko04921 | Oxytocin signalling pathway | 0.002856 | 57 |
| ko00250 | Alanine, aspartate and glutamate metabolism | 0.003097 | 19 |
| ko04080 | Neuroactive ligand-receptor interaction | 0.003467 | 127 |
| ko00020 | Citrate cycle | 0.003887 | 15 |
| ko00670 | One carbon pool by folate | 0.003959 | 11 |

Abbreviation: DEGs = differentially expressed genes.

**Supplementary Table S5**

Top 20 pathways determined using the Kyoto encyclopedia of genes and genomes analysis in meat-type ducks (E11 vs. D1).

| Pathway ID | Pathways | Adjusted *P*-value | DEGs |
| --- | --- | --- | --- |
| ko04110 | Cell cycle | 4.12E-08 | 86 |
| ko03460 | Fanconi anemia pathway | 2.45E-06 | 42 |
| ko03030 | DNA replication | 1.00E-05 | 26 |
| ko04390 | Hippo signalling pathway | 6.36E-05 | 96 |
| ko04520 | Adherens junction | 0.00012 | 51 |
| ko03440 | Homologous recombination | 0.000204 | 29 |
| ko04360 | Axon guidance | 0.000237 | 90 |
| ko00512 | Mucin type O-glycan biosynthesis | 0.000572 | 23 |
| ko05410 | Hypertrophic cardiomyopathy | 0.000644 | 51 |
| ko04310 | Wnt signalling pathway | 0.000772 | 91 |
| ko00514 | Other types of O-glycan biosynthesis | 0.000941 | 31 |
| ko05412 | Arrhythmogenic right ventricular cardiomyopathy | 0.001048 | 42 |
| ko05033 | Nicotine addiction | 0.001427 | 30 |
| ko00230 | Purine metabolism | 0.001814 | 77 |
| ko00480 | Glutathione metabolism | 0.002148 | 29 |
| ko03008 | Ribosome biogenesis in eukaryotes | 0.002981 | 52 |
| ko01100 | Metabolic pathways | 0.003035 | 709 |
| ko04512 | ECM-receptor interaction | 0.003136 | 56 |
| ko03410 | Base excision repair | 0.003598 | 21 |
| ko04391 | Hippo signalling pathway -fly | 0.004258 | 35 |

Abbreviation: DEGs = differentially expressed genes.

**Supplementary Fig. S1.** Amplification of *CHD1* gene in ducks. Agarose gel electrophoresis revealed a single band at approximately 500 bp in male embryos (Lane 1, 3, 5, 9,10) and two bands at approximately 350 and 500 bp in female embryos (Lane 2, 4, 6, 7, 8).

**Supplementary Fig. S2.** Clustering analysis of Differentially expressed genes. The redder the colour, the higher the expression level, and the greener the colour, the lower the expression level. In the heatmap, each column represents a sample and each row represents a gene. Each time (E11, E19, and D1) was clustered into its respective group, indicating that the expression patterns of differentially expressed genes within the same group were similar.
